# The Effect of Breast Size on Spinal Posture

**DOI:** 10.1007/s00266-022-03141-w

**Published:** 2022-10-24

**Authors:** Roman Michalik, Britta Kühlmann, Michael Wild, Hannah Lena Siebers, Filippo Migliorini, Jörg Eschweiler, Marcel Betsch

**Affiliations:** 1 Department of Orthopaedics, Trauma and Reconstructive Surgery, University Hospital RWTH Aachen, Pauwelsstraße 30, 52074 Aachen, Germany; 2 Department of Plastic Surgery, University Hospital Regensburg, Regensburg, Germany; 3 Department of Trauma and Orthopaedic Surgery, Klinikum Darmstadt, Darmstadt, Germany; 4 Department of Trauma Surgery and Orthopaedics, University Hospital Erlangen, Erlangen, Germany

**Keywords:** Surface topography, Spinal posture, Breast size, Back pain, Reduction mammaplasty

## Abstract

Macromastia can cause various clinical symptoms, such as low back and shoulder pain as well as sacro-iliac disorders. Because of these symptoms, some women consider breast reduction surgery. So far there does not exist a clear correlation between breast size and back pain. Purpose of this study was to evaluate if increasing breast size has a measurable effect on women’s posture using radiation free surface topography.

A total of 100 women were grouped according to their breast cup size into four groups (Cup Size: A, B, C, D). All female subjects were measured with a surface topography system, and their spinal posture and pelvic position were analysed accordingly.

Our results showed that cup size affects kyphotic angle (*p *= 0.027) and surface rotation (*p *= 0.039) significantly. Kyphotic angle increased with cup size. Multiple linear regression analysis, however, revealed that the body mass index has the greatest influence on woman’s posture, showing significant correlation to kyphotic and lordotic angle (*p *< 0.01), as to trunk (*p *< 0.01) and pelvic inclination (*p *= 0.02).

This is the first study that evaluates the influence of increasing breast size on posture using surface topography. The results match with previous studies using different measuring techniques. However, the great influence of BMI on posture is also confirmed. Therefore, in clinical practice these factors should be taken into account and be approached. Surface topography seems to be a promising tool to further investigate the influence of breast size on posture.

*Level of Evidence IV* This journal requires that authors assign a level of evidence to each article. For a full description of these Evidence-Based Medicine ratings, please refer to the Table of Contents or the online Instructions to Authors www.springer.com/00266.

**Supplementary Information:**

The online version contains supplementary material available at 10.1007/s00266-022-03141-w.

## Introduction

Women with macromastia can suffer from back, shoulder and neck pain caused by changes in spinal posture [1–5]. These postural changes might be triggered by a shift of the body´s centre of gravity due to large breasts, potentially resulting in an increase in the thoracic kyphosis combined with compensatory changes of the cervical and lumbar spine [1, 6, 7].

In patients with persistent and therapy resistant back pain caused by macromastia, mammoplasty surgery seems to be a therapeutic option to relieve back pain [5]. However, most health insurance companies do not cover the cost of mammoplasty surgery in patients with back pain. Prior studies have tried to evaluate correlation between breast size, back pain and postural changes. A study by Lapid et al. using standardized lateral photographs, examined changes of spinal posture pre- and postop in forty-two patients, who underwent breast reduction surgery. The authors found a significant decrease in the trunk inclination angle after mammoplasty compared to a control group [3]. A study by Berberoğlu *et al.* showed that mammoplasty may lead to a significant improvement in spinal posture examined by radiographs with a significant correlation between the amount of excised breast tissue volume and the decrease in neck, back and lumbar pain [4]. Coltman et al. tried to establish that women with macromastia, lower age and a greater nipple-to-nipple distance suffer from more musculoskeletal pain using a Flexicurve ruler for measurement of thoracic kyphosis [8]. The problem with most these studies is that they have tried to quantify spinal posture by measuring the position of bony structures using ionizing radiation or by semi-quantitative methods such as lateral photographs or rulers, which are known to have limited validity and reliability [9, 10].

With surface topography, an innovative measuring system is available, which provides a valid and reliable examination not only of spinal posture, but also of the underlying spine [11–15]. This technique works by projecting light-line patterns on the back surface and can therefore be used even in pregnant women and adolescent patients repeatedly [16, 17].

Purpose of this study was to use this innovative technique to establish a correlation between breast size and changes in spinal posture in women. We hypothesized that women with macromastia will show an increase in thoracic kyphosis compared to women with small breast sizes.

## Materials and Methods

A total number of 100 women were enrolled in this study to measure the effects of breast size on spinal posture. The human subjects research review board approved the study protocol of this study (3310). All female volunteers were informed about the study, gave their oral and written consent and were given the option to discontinue participation at any time. Women without any history of lower extremity, pelvic and spine fractures or vertebral diseases were included in this study. Female volunteers younger than 18 years, postmenopausal and pregnant women were excluded from this investigation. For each of the women underband and overbust measurements, breast circumference as well as age, height and weight was measured. The body mass index (BMI) of all participants was calculated with the formula: ${\text{BMI}} = \frac{{{\text{weight}}}}{{{\text{height}}^{2} }}\;{\text{in}}\;\left[ {\frac{{{\text{kg}}}}{{{\text{m}}^{2} }}} \right]$. The overall demographic data of the study group are listed in Table 1.

**Table 1:** Overall demographics of the evaluated subjects in this study with mean and standard deviation (±) listed for each parameter

N	Age (years)	Height (cm)	Weight (kg)	BMI (kg/m^2^)	Breast circumference (cm)	Cup size (A=1, D=4)
100	31.98 ± 11.83	168.02 ± 6.57	66.97 ± 12.28	23.70 ± 3.99	92.10 ± 13.60	2.47 ± 1.11

To evaluate for the effects of breast size on the spinal posture in women, all volunteers were divided into four groups according to their breast cup size: (1) A Cup, (2) B Cup, (3) C Cup, (4) D Cup. All cup sizes were determined by calculating the difference between the overbust and underband measurements: A Cup (< 6.5 cm), B Cup (6.5–13 cm), C Cup (13 − 19.5 cm), D cup (> 19.5 cm). Equal group sizes were established with 24 women with cup size A, 29 (B), 23 (C), and 24 (D). For clinical evaluation, we used the Oswestry low back pain questionnaire [18] to evaluate and quantify the disability caused by low back pain.

To be able to measure the influence of breast size on the spinal posture and pelvic position without any harmful X-ray radiation, we decided to use a validated optical, surface topography system (Formetric^®^, Diers International GmbH, Schlangenbad, Germany). A single female physician measured all women, and the measurements were performed with the women standing in a relaxed posture with extended knees and arms hanging to the sides.

**Fig. 1:**
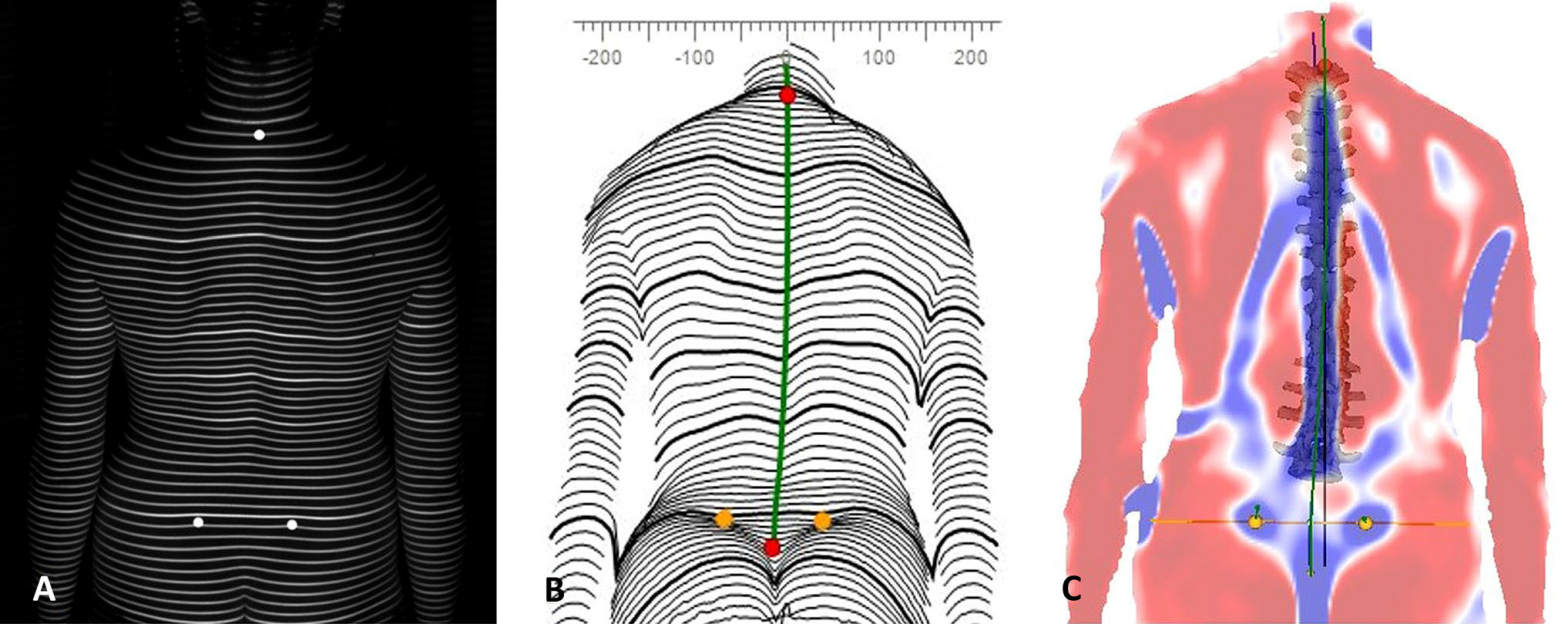
Unclothed back of patient is floodlit by the projector of the surface topograph to project horizontal parallel light lines (**A**). A surface reconstruction of the back is performed by transforming the lines and their corresponding curvature into a three-dimensional scatter plot (**B**, **C**).

Surface topography, developed in the 1980s by Drerup and Hierholzer, is a method for stereophotogrammetric surface measurements of the back [19]. It uses a slide projector to project horizontal parallel light lines onto the unclothed back surface of a patient. A surface reconstruction of the back is performed by transforming the lines and their corresponding curvature into a three-dimensional scatter plot (Figure 1A–C).

**Fig. 2:**
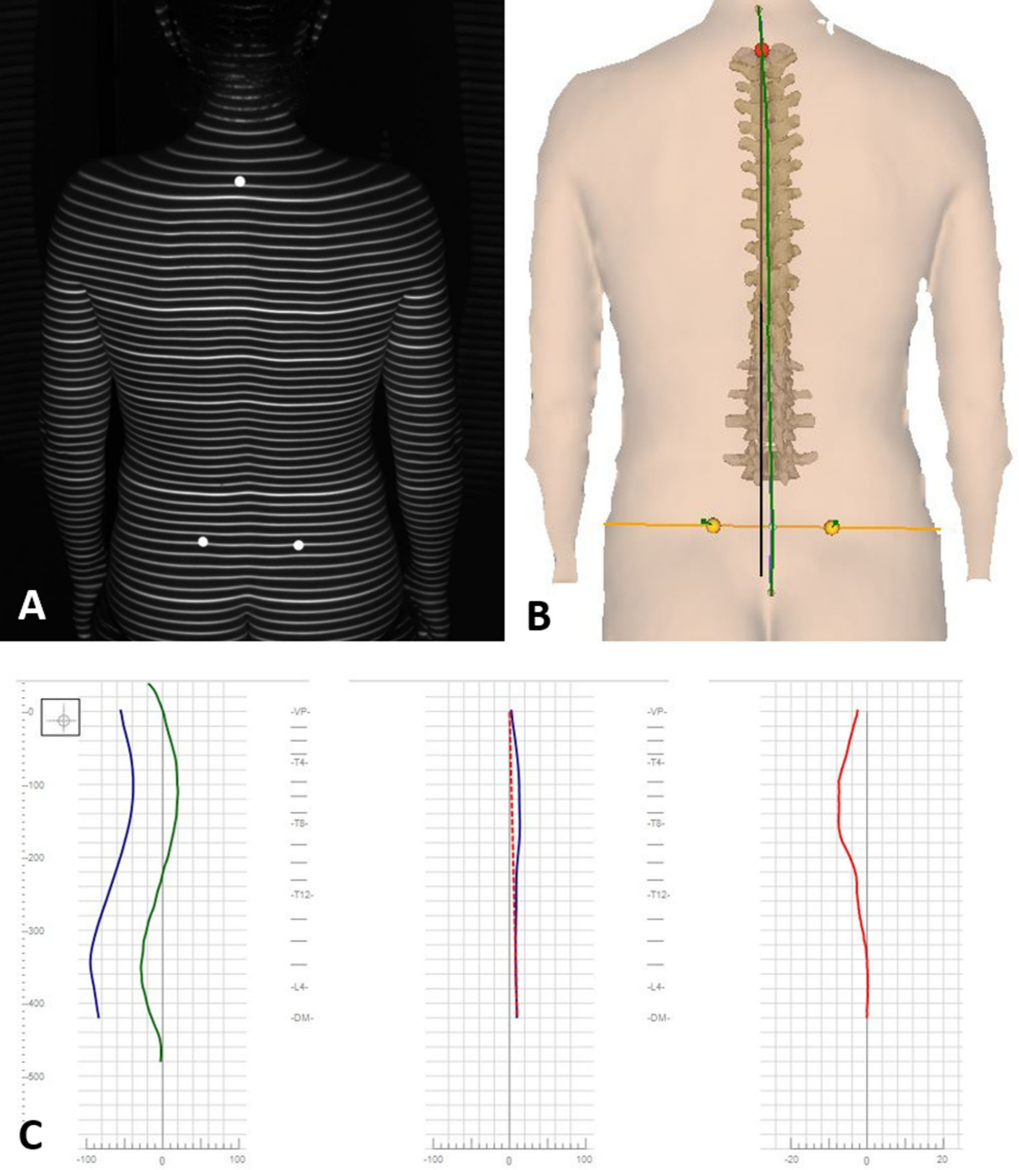
Surface topographic imaging are automatically processed to create a 3D-model that allows an accurate 3D reconstruction of the subject´s spinal column (**A**, **B**). Further analysation and measurements (for example deviation in frontal or sagittal plane) are done automatically by the system (**C**).

A 3D-model of the spine can then be calculated based on the specific convex shape of the spinous process of the vertebra prominence (VP) and the concavity of the lumbar dimples, which can be localized by the system with an accuracy of ± 1mm [13, 14]. The 3D-model was developed by Turner-Smith and Drerup, Hierholzer and is correlated with over 500 reference radiographs of the spine allowing an accurate 3D reconstruction of the subject´s spinal column from the topographic image taken [20, 21] (Figure 2). Studies of reproducibility found that intrarater reliability was high (Chronbach’s Alpha from 0.921 to 0.992) as was the interrater reliability (Chronbach’s Alpha 0.979) [22, 23] and its high validity was proved in a recent meta-analysis [24].

The following spinal and pelvic parameters were measured and evaluated for this study. The kyphotic angle is defined as the angle between the surface tangents on points between the cervical-thoracic (ICT) and thoraco-lumbar (ITL) transition. The lordotic angle is the angle between the surface tangents between ITL and the lumbo-sacral transition (ILS).

The angle between the connecting line of VP-DM and a vertical external line of gravity is defined as the trunk inclination. A further parameter analysed was the lateral deviation of the spine, which is defined as the deviation of the spinal midline from the plum line between the VP and DM in the frontal plane. Surface rotation is defined as the value of the horizontal components of the surface normal on the line connecting the spinous processes of the spine (symmetry line). Finally, pelvic inclination is the mean vertical torsion in degrees of the two surface normals on the two lumbar dimples. The surface topographic system, protocol and parameters have been used and proved in numerous prior studies [11, 12, 25–28].

### Data Analysis

Normality of the data was assumed based on the inspection of histograms and q–q plots. Unifactorial ANOVA (Tukey HSD test for post hoc multiple comparisons) was used to assess for differences in the spinal and pelvic parameters between different groups. The level of significance was set at *p*<0.05.

To analyse different impact variables, a multiple linear regression model was calculated for each posture parameter. Independent variables were the cup size, breast circumference, BMI, age, and the OSWESTRY Disability Score. We analysed the quality of the model and the variables with a significant correlation to the posture parameters. Requirements for multiple linear regression were checked. Based on the results, one participant was excluded and the analysis was repeated. Statistical analysis and graphic presentations were prepared using software SPSS 25.0^®^ (SPSS Inc., Chicago, USA).

## Results

Our results showed a significant effect of cup size on the thoracic kyphotic angle (*p *= 0.027) and surface rotation (*p *= 0.039). All other parameters were not significantly influenced by cup size (Table 2).

**Table 2:** Effect of cup size on pelvic and spinal parameters were calculated using ANOVA testing

Parameter	Kyphotic angle	Surface rotation	Lordotic angle	Lateral deviation	Trunk inclination	Pelvic tilt	Pelvic torsion	Pelvic inclination
Effect by cup size (*p*) overall	**0.027**	**0.039**	0.304	0.115	0.755	0.415	0.826	0.646

Kyphosis (P = 0.027) and surface rotation (P = 0.039) are significantly influenced by cup size and were further analysed using post hoc testing (Table 3)

**Fig. 3:**
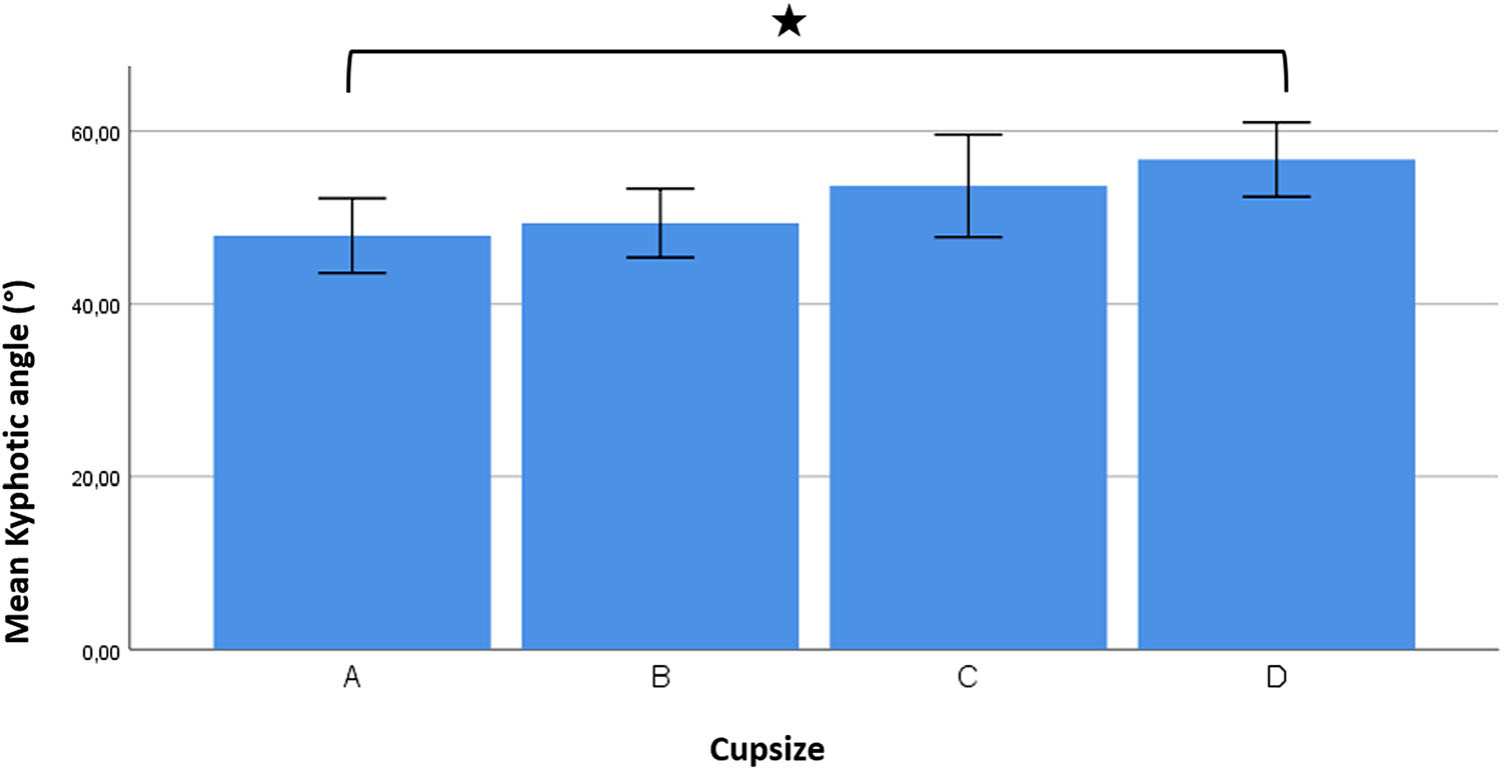
With increasing cup size, we did find an increase in the kyphotic angle. In women with cup size A, we measured a mean kyphotic angle (mean value ± SD) of 47.9° ± 10.3°, with cup size B of 49.3° ± 10.5°, with cup size C of 53.9° ± 13.5° and with cup size D of 56.7° ± 10.2°. The increase in kyphosis was significant (*p *= 0.037) between women with cup size A and D.

Kyphotic angle increased continuously with increasing cup size (Fig. 3). Post hoc testing showed a significant difference of the thoracic kyphosis in between subjects with cup size A and D (*p *= 0.037), whereas no significant differences between groups were found regarding the surface rotation (Table 3).

**Table 3:** Post hoc testing using Tukey HSD test shows a significant difference of the kyphosis in between subjects with cup size A and D (*p *= 0.037)

Post hoc analysis	Kyphotic angle	Surface rotation
A versus B	0.966	0.066
A versus C	0.297	0.066
A versus D	**0.037**	0.678
B versus C	0.515	0.999
B versus D	0.087	0.548
C versus D	0.786	0.507

Significant differences in surface rotation between cup size groups were not calculated (*p *= 0.066–0.999)

As a next step, we aimed to evaluate which patient specific parameter has the greatest influence on spinal posture.

The performed multiple linear regression analysis revealed a significant predictability based on the variable cup size, age, OSWESTRY Score, BMI and breast circumference for the parameters kyphotic and lordotic angle (*p *< 0.001) as well as trunk (*p *< 0.001) and pelvic inclination (*p *= 0.005). Further analysis showed that BMI is a significant predictor for all these parameters (*p *< 0.001), while cup size is only a significant predictor for lordosis (*p *= 0.035). The influence of further variables is found in Table 4.

**Table 4:** Multiple linear regression analysis is used to evaluate the influence of cup size, age, Oswestry Index, BMI and breast circumference (independent variables) on posture parameter (dependent variables)

Variables	Kyphotic angle			Lordotic angle			Trunk inclination			Pelvic inclination		
*p*-value; Regression coefficient α; β	*p*	*α*	*β*	*p*	*α*	*β*	*p*	*α*	*β*	*p*	*α*	*β*
Cup size	.791	.257	.025	**.035**	− 1.881	− .215	.244	− .317	− .124	.274	− .612	− .121
Age	**.007**	.248	.253	.716	.029	.036	**.017**	− .061	− .254	**.048**	− .103	− .217
Oswestry	.055	− .241	− .173	.413	.093	.080	.379	.031	.090	**.002**	.230	.342
BMI	**< .001**	1.259	.434	**<.001**	1.264	.522	**<.001**	.362	.511	**.020**	.415	.296
Breast circ.	.190	.121	.142	.582	.046	.064	.740	.009	.041	**.049**	− .105	− .255

Significant correlation of BMI was shown for multiple parameters as cup size, breast circumference and age only revealed to correlate with single parameters (printed in bold type)

## Discussion

This is the first study using surface topography to evaluate a correlation between breast size and spinal posture. The results of our study show that the thoracic kyphotic angle and the surface rotation are significantly influenced by breast cup size. In the examined population, the kyphotic angle increases with greater cup sizes and a significant difference between subjects with cup size A and D was found. Multiple linear regression analysis, however, showed that cup size is a significant predictor for the lumbar lordosis only. Women’s BMI revealed to be a significant predictor for posture regarding kyphotic and lordotic angle, trunk a pelvic inclination.

A larger breast size can lead to changes in posture, neck problems and back pain which are also the main reasons for woman to undergo reduction mammoplasty [29, 30].

Mammoplasty has been proved to be a safe and effective surgical technique, and it is therefore widely used as a surgical option to treat musculoskeletal discomfort caused by macromastia [31]. Studies showed that the spinal posture is related to the breast size of patients and that mammoplasty can lead to improved spinal posture associated with a pain reduction [32]. Despite these findings, some health insurance companies regard breast reduction surgery as purely aesthetic and do not grant cost coverage [1].

Former studies using radiographs indicated that large breasts may induce changes in thoracic kyphosis and lumbar lordosis angles [1, 2, 6], which supports the findings of our present study. Using light-based surface topography, our results show an increase in thoracic kyphosis with larger cup size. The kyphotic angle was significantly higher in woman with cup size D compared to those with Cup size A (A: 47.9° ± 10.3°, D of 56.7° ± 10.2°). Studies by Coltman et al. evaluating the effect of breast size on the upper torso did show a higher thoracic kyphosis in woman with larger breasts [7, 33]. Further work of this study group revealed an association between breast size and thoracic musculoskeletal pain [8]. Besides the predominant effects on the thoracic spine caused by large breasts, there has been radiologically shown some effects on the lumbar lordosis as well [1, 2]. A study by Findikcioglu et al. found a significantly higher lumbar lordosis in woman with a cup size D compared to those with an A cup using X-rays [1]. The significant effect on the lumbar lordosis by cup size was also shown by multiple linear regression analysis in our study.

While on the one hand X-ray-based studies do only take the skeletal system into account and use ionizing radiation, other so far performed studies evaluating the musculoskeletal system have used semiquantitative methods with low validity and reliability [9, 10]. In this study, we used an optical and surface topography system (Formetric^®^, Diers International GmbH, Schlangenbad, Germany), which has been validated for measuring posture in preliminary studies [11, 12, 24–28, 34, 35]. We were able to detect the effect of increasing cup size on woman’s posture. As shown in the performed linear regression analysis, there is a significant effect of BMI on posture in our study population. Macromastia is often associated with increased BMI and obesity, which can also influence the posture. A study by Goulard *et al.* using lateral photographs for posture analysis showed that reduction mammoplasty, which did not affect the pre- and postoperatively measured BMI, improved body posture and the alignment of the shoulders, trunk and pelvis [36]. Similar results were published by Coltman et al. showing a significant association between cup size, respectively, breast size and posture but as well an association of spinal posture with BMI [33]. These findings may lead to the conclusion that weight reduction should be considered the first line strategy to improve spinal posture before undergoing mammoplasty surgery.

Several studies have been conducted to evaluate the influence of weight and obesity on posture.

Lang-Tapia et al. conducted a study in 297 women and 362 men showing that overweight and obese patients have significantly less lumbar lordosis and more thoracic kyphosis compared with non-overweight subjects [37]. In contrast, a recent meta-analysis by Molina-Garcia et al including 1,757,107 children and adolescents to study the impact of childhood obesity on joint alignment in general found a correlation between obesity and lumbar hyperlordosis as well as genu valgus, flatfoot deformity when compared with a control group [38].

In previous research, it was also tried to establish a correlation between back pain and spinal posture. However, the majority of these studies evaluating the correlation of back pain with breast size used either ionizing radiation, e.g. X-rays, or measurement techniques with limited variability and reliability to examine the spinal posture. The surface topography, which has been used successfully in the present and numerous prior studies, benefits from a high validity and reliability compared to X-ray and the posture analysation of the whole musculoskeletal system including soft tissue. Proving the feasibility in analysation of breast size associated posture changes, the surface topography approach can further be used as a valuable optional tool in this field. The system allows the analysation of pelvic and spinal posture during walking as well. Regarding the effect of breast size on posture and pain, additional symptoms and biomechanical aspects come in place under dynamic conditions. The evaluation of proper support of women’s breasts under dynamic conditions and associated posture are further planned studies. Recent developments allow a 360-degree reconstruction of the human torso, which might be a promising tool for simultaneous breast and posture analysis.

Overall, the wide range of applications in analysing posture by surface topography should be taken into account in clinical practice and be part of further research.

### Limitations

Our study is a first step to possibly identifying posture problems concerning breast size with a radiation-free surface topography system. While the system has proven high reliability and validity, additional radiographic imaging of our study group was not performed. Further limitations of the study are the relatively small number of subjects, lack of diversity, young age and the low level of back pain. Further, it needs to be mentioned that surface topography relies on the detection of anatomic landmarks that might be impaired due to overlaying soft tissues in obese patients (BMI > 35 kg/cm^2^). With surface topography, these anatomical landmarks can be automatically or manually detected. A study by Knott et al. found no strong correlation between surface topography parameters and the patients’ BMI in a population with BMIs between 16.9 and 29 [39]. In our study, the average BMI was 23.70 ± 3.99 (kg/m^2^) and problems with the automated fixpoint detection were not noticed. As a fall-back solution, there is the possibility to use reflective markers to increase accuracy of 4D rasterstereography in certain cases as it might be necessary in patients with BMI > 30 kg/m^2^.

## Conclusion

Surface topography is a valuable tool to measure spinal posture and pelvic position without harmful radiation in women with back pain and macromastia. This technique will help in future studies to further examine the effects of mammoplasty surgery on the spinal posture. Breast size seems to have the greatest effect on the lumbar lordosis of women and kyphotic angle increased continuously with increasing cup size. However, it must also be noted that from all patient specific factors analysed, the BMI had the greatest influence on women’s posture.

### Supplementary Information

Below is the link to the electronic supplementary material.

Supplementary file1 (DOCX 15 KB)

## Data Availability

The data that support the findings of this study are available from Marcel Betsch and Roman Michalik, but restrictions apply to the availability of these data, which were used under license for the current study and so are not publicly available.
