# Supplementary material for: The Effect of Breast Size on Spinal Posture
Source: Aesthetic Plast Surg. 2022 Oct 24;48(7):1331–8. doi: 10.1007/s00266-022-03141-w (PMC11035396; doi:10.1007/s00266-022-03141-w)
Supplement: Supplementary file 1 — Supplementary file1 (DOCX 15 KB) [file 266_2022_3141_MOESM1_ESM.docx]

Table 4: Multiple linear regression analysis is used to evaluate the influence of cup size, age, Oswestry Index, weight, height and breast circumference (independent variables) on posture parameter (dependent variables). Significant corelation of weight was shown for multiple parameters as cup size, breast circumference and age only revealed to correlate with single parameters (printed in bold type).

| ***Variables*** | **Kyphotic angle** | | | **Lordotic angle** | | | **Trunk inclination** | | | **Pelvic inclination** | | |
| --- | --- | --- | --- | --- | --- | --- | --- | --- | --- | --- | --- | --- |
| ***p-value; Regression coefficient* α ;β** | **p** | **α** | **β** | **p** | **α** | **β** | **p** | **α** | **β** | **p** | **α** | **β** |
| **Cup size** | .847 | .186 | .018 | **.029** | -1.935 | -.221 | .218 | -.334 | -.130 | .273 | -.613 | -.121 |
| **Age** | **.006** | .252 | .257 | .694 | .032 | .039 | **.017** | -.061 | -.253 | **.039** | -.107 | -.227 |
| **Size** | .158 | -.226 | -.128 | .067 | -.268 | -.182 | .068 | -.082 | -.191 | .058 | -.176 | -.207 |
| **Weight** | **<.001** | .487 | .516 | **<.001** | .479 | .608 | **.001** | .138 | .599 | **.023** | .147 | .323 |
| **Oswestry** | .079 | -.223 | -.160 | .368 | .103 | .089 | .350 | .033 | .097 | **.003** | .218 | .325 |
| **Breast circ.** | .457 | .073 | .086 | .913 | .010 | .014 | .959 | -.001 | -.007 | .090 | -.097 | -.235 |
